# Breeding, Early-Successional Bird Response to Forest Harvests for Bioenergy

**DOI:** 10.1371/journal.pone.0165070

**Published:** 2016-10-25

**Authors:** Steven M. Grodsky, Christopher E. Moorman, Sarah R. Fritts, Steven B. Castleberry, T. Bently Wigley

**Affiliations:** 1 North Carolina State University, Department of Forestry and Environmental Resources, Fisheries, Wildlife, and Conservation Biology Program, Box 7646, Raleigh, NC 27695, United States of America; 2 Texas Tech University, Department of Natural Resources Management, P.O. Box 42125, Lubbock, Texas, 79409, United States of America; 3 University of Georgia, Warnell School of Forestry and Natural Resources, 180 E. Green St., Athens, GA 30602, United States of America; 4 National Council for Air and Stream Improvement, Inc., P.O. Box 340317, Clemson, SC 29634, United States of America; Sichuan University, CHINA

## Abstract

Forest regeneration following timber harvest is a principal source of habitat for early-successional birds and characterized by influxes of early-successional vegetation and residual downed woody material. Early-successional birds may use harvest residues for communication, cover, foraging, and nesting. Yet, increased market viability of woody biomass as bioenergy feedstock may intensify harvest residue removal. Our objectives were to: 1) evaluate effects of varying intensities of woody biomass harvest on the early-successional bird community; and (2) document early-successional bird use of harvest residues in regenerating stands. We spot-mapped birds from 15 April– 15 July, 2012–2014, in six woody biomass removal treatments within regenerating stands in North Carolina (*n* = 4) and Georgia (*n* = 4), USA. Treatments included clearcut harvest followed by: (1) traditional woody biomass harvest with no specific retention target; (2) 15% retention with harvest residues dispersed; (3) 15% retention with harvest residues clustered; (4) 30% retention with harvest residues dispersed; (5) 30% retention with harvest residues clustered; and (6) no woody biomass harvest (i.e., reference site). We tested for treatment-level effects on breeding bird species diversity and richness, early-successional focal species territory density (combined and individual species), counts of breeding birds detected near, in, or on branches of harvest piles/windrows, counts of breeding bird behaviors, and vegetation composition and structure. Pooled across three breeding seasons, we delineated 536 and 654 territories and detected 2,489 and 4,204 birds in the North Carolina and Georgia treatments, respectively. Woody biomass harvest had limited or short-lived effects on the early-successional, breeding bird community. The successional trajectory of vegetation structure, rather than availability of harvest residues, primarily drove avian use of regenerating stands. However, many breeding bird species used downed wood in addition to vegetation, indicating that harvest residues initially may provide food and cover resources for early-successional birds in regenerating stands prior to vegetation regrowth.

## Introduction

Early-successional vegetation structure ephemerally occurs during forest succession and is precipitously declining in North America due to changes in natural and anthropogenic disturbance regimes [1]. As such, conservation of disturbance-dependent plant and animal species specifically adapted to early-successional vegetation has grown in priority [2, 3, 4, 5]. Indeed, some early-successional species are imperiled, spanning from the Karner blue butterfly (*Lycaeides melissa samuelis*) to the New England cottontail (*Sylvilagus transitionalis*). Birds especially are useful ecological indicators of land-use change for early-successional communities and the species therein because numerous bird species are restricted to particular stages of forest stand development, sensitive to changes in forest structure, and exhibit responses to disturbance indicative of general patterns for most other organisms [1, 6]. Early-successional birds are adapted to open-canopy conditions created by increasingly uncommon natural and anthropogenic disturbances [1, 7, 8] and presently rank among the highest conservation priority avian habitat specialists [2, 9].

Currently, forest regeneration following timber harvest is a principal source of habitat for early-successional birds [1], especially in regions supporting forest industry like the southeastern United States [9]. Indeed, intensively managed pine (*Pinus* spp.) forests account for approximately 20% of forest cover in the region, with 13.4 million ha in loblolly pine (*Pinus taeda*) production alone [10, 11]). Overstory tree removal following a clearcut harvest increases light levels reaching the forest floor, which in turn stimulates development of a dense layer of herbaceous and soon after, woody, early-successional vegetation [12, 13, 14]. Importantly, clearcutting also can result in habitat patches large enough to accommodate area-sensitive, early-successional bird species [15, 16]. The positive relationship between vegetation structure and diversity following clearcutting and early-successional birds has been well studied, and young, intensively managed forests have been shown to provide habitat for early-successional birds prior to canopy closure [9, 17, 18, 19].

Following timber harvest, the influx of early-successional vegetation is accompanied by substantial increases in downed wood from harvest residues [20]. Volume of downed wood often follows a U-shaped timeline, with large volumes immediately created after a disturbance, followed by a steady decrease in volume as downed wood decays, and then a slow increase in volume as mature trees senesce and die [20, 21]. Downed wood plays a major role in forest ecosystem function and integrity [22, 23]. In particular, downed wood provides important food and cover for wildlife, including early-successional birds [24, 25]. Early-successional birds may use downed wood for communication, cover, foraging, and nesting in regenerating stands [24, 26, 27].

Increases in the market viability of woody biomass as a forest bioenergy feedstock could lead to intensified levels of harvest residue removal [23]. Forests are capable of producing vast amounts of woody biomass (e.g., harvest residues), which in turn may be used in forest bioenergy production to generate heat, electricity, and biofuels [28, 29]. Currently, harvest residues are an important feedstock for production of wood pellets [30], co-generated electricity (i.e., coal and woody biomass simultaneously burned; [31]), and, to a lesser extent, liquid transportation biofuels [32, 33].

Woody biomass harvests often occur following clearcutting in intensively managed forests of the southern United States [23]. Currently, the region is the largest exporter of wood pellets in the world [34], and wood pellet production in the region is predicted to increase [33]. The region also is experiencing more rapid development of forest bioenergy-production facilities (e.g., woody biomass power plants) than anywhere else in the world [35, 36]. Nearly 50% of second generation biofuels required to meet United States biofuel mandates by 2022 is projected to be supplied by forests of the southeastern United States [37].

Concerns regarding potential effects of woody biomass harvests on forest ecosystems have led to development of non-regulatory biomass harvesting guidelines (BHGs) by some state agencies and non-profit organizations in regions supporting woody biomass harvests (*see* [38] for Southeast BHGs). BHGs specify target volumes of downed wood to be retained on the forest floor for wildlife habitat, nutrient cycling, and erosion control [38, 39]. In general, BHGs are based on the assumption that “more” downed wood is better than “less” downed wood (e.g., [40]). However, suggested volumes and spatial arrangements of downed wood vary among regional BHGs, and BHGs have limited technical underpinnings due to a paucity of empirical support. Furthermore, BHGs will need to be better justified in scientific literature to offset stakeholder perceptions that they may create unnecessary costs [41]. As such, research is needed to determine effects of woody biomass harvests and implementation of BHGs on forest ecosystem sustainability and wildlife habitat.

Few studies have specifically addressed relationships between birds and downed wood, and no studies have addressed early-successional, breeding bird response to operational-scale woody biomass harvests [23, 24], suggesting that studies of early-successional bird response to harvest residue retention following woody biomass harvests are warranted. Thus, our primary objectives were to: (1) evaluate effects of varying intensities of woody biomass harvest on the early-successional bird community; and (2) document early-successional bird use of downed wood in regenerating stands.

## Methods

### Study area and design

We examined breeding birds in eight replicate regenerating stands (hereafter “blocks”) in intensively managed loblolly pine (*Pinus taeda*) plantations within the Coastal Plain Physiographic Region of the southeastern United States. Our study included four blocks [70.5 ± 6.1 (mean ± SE) ha] in Beaufort County, North Carolina (NC) and four blocks (64.64 ± 3.1 ha) in Georgia (GA): three in Glynn County, GA and one in Chatham County, GA. All research was conducted on private forest industry property with permission from the landowners. In NC, blocks were managed for sawtimber production, commercially thinned twice prior to harvest, and clearcut harvested at 32–39 years old. In GA, blocks were managed for chip-and-saw and pulpwood production, commercially thinned once at each Glynn County block and twice at the Chatham County block prior to harvest, and clearcut harvested at 25–33 years old. Soils at the NC blocks predominantly were loam and silt loam, while soils at the GA blocks mainly were loam, clay loam, and fine sandy loam.

Following clearcut harvest in 2010–2011, we implemented woody biomass removal treatments (herein “treatments”) at each block. We used a randomized complete-block experimental design, dividing each block into the following six, adjacent treatments: (1) clearcut with a traditional woody biomass harvest and no biomass harvesting guidelines implemented (NOBHGs); (2) clearcut with 15% retention of harvest residues evenly dispersed throughout the treatment (15DISP); (3) clearcut with 15% retention of harvest residues clustered in large piles throughout the treatment (15CLUS); (4) clearcut with 30% retention of harvest residues evenly dispersed throughout the treatment (30DISP); (5) clearcut with 30% retention of harvest residues clustered in large piles throughout the treatment (30CLUS); and (6) clearcut with no woody biomass harvest (i.e., clearcut only; NOBIOHARV), which served as a reference. In NC, treatments averaged 11.7 ± 0.5 (mean ± SE) ha and ranged from 8.4–16.3 ha; in GA, treatments averaged 10.7 ± 0.4 ha and ranged from 7.6–14.3 ha. We defined harvest residues as non-roundwood stems and pine tops and limbs traditionally considered non-merchantable prior to the advent of forest bioenergy-driven woody biomass markets.

In each treatment, all standing pines merchantable as roundwood were cut and transported to a logging deck with a grapple skidder. For the NOBHGs treatments, we instructed loggers to glean all harvest residues they deemed merchantable as woody biomass. For the NOBIOHARV treatments, pine roundwood was harvested; however, we instructed loggers to fell and leave all other stems (i.e., primarily midstory hardwoods) not harvested as roundwood and pine tops and limbs in homogenously distributed skidder grapple loads throughout each NOBIOHARV treatment.

To implement the four treatments emulating BHGs, we used ArcGIS (ESRI, Redlands, California, USA) to delineate retention areas that represented either 15% or 30% of the total treatment area. Prior to clearcut harvest, we located retention areas using a hand-held Garmin Rino global positioning system (Olathe, Kansas, USA) and flagged boundaries. We retained all hardwoods not merchantable as roundwood and other pine residues in retention areas. Retention areas were clearcut after loggers harvested 85% or 75% of the stand, and harvest residues from retention areas were redistributed throughout the treatment unit with a grapple skidder. Loggers spread retained harvest residues from retention areas evenly throughout the DISP treatments or in randomly placed piles throughout the CLUS treatments. Because we created treatments by distributing harvest residues with a grapple skidder, individual piles of harvest residues in the CLUS and NOBIOHARV treatments were approximately the size of one grapple load (volume = 36.19 m^3^/ha^-1^; [42]). Harvest residues from the non-retention areas and the entire NOBHG treatment were chipped at the logging deck during harvest.

Although treatment implementation was similar in NC and GA, site preparation differed between states. In NC, site preparation occurred following clearcut harvest and implementation of treatments in the winter of 2010–2011. Blocks were sheared using a V-shaped blade, bedded into continuous, mounded strips of soil (i.e., beds) approximately 3 m wide and < 1 m tall, and planted with loblolly pine during the fall-winter of 2011–2012 at a density of ≈1100 trees ha^-1^. Shearing moved retained harvest residues into the 3-m space between pine beds (i.e., interbeds). Consequently, woody biomass was rearranged following shearing into long, linear rows in interbeds parallel to pine beds; however, volume of harvest residues was largely unaltered by shearing [42]. Blocks were treated with the following two post-harvest herbicide applications of Chopper (BASF, Raleigh, North Carolina, USA) for herbaceous weed control: (1) a broadcast application (applied by helicopter) one year after clearcut harvest; and (2) a banded application (applied only to pine trees in bedded rows) two years after clearcut harvest.

In GA, most harvest residues in treatments were concentrated into large, linear piles (i.e., windrows) extending for the entire length of treatments or into large, conical piles (1–100 m^3^) within treatments. As such, few individual stems and no small downed wood piles (<1 m^3^) occurred between windrows (~30–50 m apart) in treatments. In Glynn County (GA), two blocks were bedded in the summer of 2011 and the remaining block was bedded in fall 2011. All Glynn County (GA) blocks were planted in winter 2012 at a density of ≈1495 trees ha^-1^ and treated with Arsenal (BASF, Raleigh, North Carolina, USA) and sulfometuron methyl for herbaceous weed control one year after clearcut harvest. In 2012, the Chatham County (GA) block was bedded and planted at a density of ≈726 trees ha^-1^ and received a broadcast treatment of Chopper one year after clearcut harvest.

In NC, all blocks and treatment plots were bordered by drainage ditches (~1 m wide) containing similar vegetation which was unaffected by site preparation and thus more developed than vegetation growing in treatments. In both states, a logging road (~3.7 m wide) separated most blocks from adjacent forest stands, which typically fell into two age classes: 1) young loblolly pine stands (~10 years old); and 2) mature loblolly pine stands (~30 years old). Snags were rare in all NC blocks and occurred sporadically throughout GA blocks. In GA, blocks contained some retained, riparian forests (e.g., stringers) that were unharvested during clearcutting.

### Avian sampling

In NC and GA, we surveyed breeding birds from 15 April– 15 July during the 2012–2014 breeding seasons by spot-mapping [27, 43]. Blocks were traversed along uniformly distributed, continuous strip transects in each treatment plot. Transects were 25 m from all block boundaries, were 50 m apart from one another, and ran the entire length of the blocks and treatments. We surveyed birds no further than 25 m to either side of transect lines throughout treatments. For each survey, one experienced observer continuously walked along transects within each treatment and recorded the spatial location of each bird heard or seen on maps of treatments. For each detected bird, the observer recorded the following information: 1) discernable behavior (e.g., counter-singing, perching, foraging); 2) movement after initial detection (i.e., with directional arrows on maps), which aided in minimizing double-counting of individuals; 3) whether the bird was on the ground within ~1 m of a downed wood pile or windrow (herein “near pile”), within a downed wood pile or windrow (herein “in pile”), on branches protruding from a downed wood pile or windrow (herein “on branch of pile”), or among vegetation of any type (i.e., not in harvest residues or on bare ground); and 4) estimated distance to the nearest drainage ditch (i.e., edge) based on one of three classes: 1) 0–25 m; (2) 25–50 m; and (3) and ≥ 50 m. Because we surveyed breeding birds only within 25 m to either side of transect lines in relatively open areas consisting primarily of low-lying grasses and forbs and sparsely distributed shrubs, we assumed detection probability in treatments was near 100% [44, 45]. We did not handle birds during avian sampling; as such, our study was exempt from approval by the North Carolina State University Institutional Animal Care and Use Committee and federal/state agencies.

Observers conducted one survey of all treatments in one entire block between sunrise and 1000 hours on mornings with no precipitation and winds ≤25 km/hour. Observers started each survey route at a random corner of each block and alternated the order in which each block was surveyed to eliminate potential temporal and directional biases. In NC, two observers independently surveyed blocks and treatments therein at the following annual frequencies: 1) two blocks 13 times and two blocks 12 times in 2012; 2) two blocks 9 times, one block 8 times, and one block 7 times in 2013; and 3) all blocks 8 times in 2014. In GA, two observers independently surveyed blocks and treatments therein at the following annual frequencies: 1) three blocks 12 times and one block 5 times in 2012; 2) one block 22 times, two blocks 21 times, and one block 10 times in 2013; and 3) one block 16 times, two blocks 15 times, and one block 5 times in 2014.

We used spatiotemporally explicit locations of singing, male birds derived from our spot-mapping surveys to delineate territories in treatments. Our method of territory mapping passerines was appropriate because most species consistently sing and conspicuously maintain and defend territories throughout the breeding season [43]. We defined a territory as a cluster of at least three detections recorded during independent visits throughout an individual breeding season. We used observed movements and counter-signing to more accurately delineate territories. We calculated territory density in each treatment as total number of individual territories divided by treatment area (ha) and converted these densities to territories/40 ha to facilitate interpretation. In <10% of cases, individual territories spanned across two treatment boundaries. When this occurred, we calculated the proportion of the territory in each treatment based on the number of detections recorded in each treatment (e.g., two detections in treatment = 0.66, one detection in adjacent treatment = 0.33) and incorporated these proportions into our territory density calculations [46].

### Quantifying harvest residues

We measured scattered and piled downed wood in each treatment at the NC and GA blocks using the line-intersect sampling (LIS) technique [47] and a visual encounter method (*see* [42] for detailed methods). In NC, we located each pile of downed wood in each treatment, measured its length, width, and height, and visually estimated its packing ratio (i.e., density of wood in pile; 0–100%). In the GA treatments, we measured the width, height, and visually estimated packing ratio every 50 m along each windrow and at each spot-pile. Because windrows often ran the entire length of treatments, we measured length of each windrow in ArcGIS using post-harvest aerial imagery (Google Maps, Mountain View, California). For both states, we summed volume of piled downed wood estimated from the visual encounter method and volume of scattered downed wood estimated using the LIS method to generate total volume of harvest residues (m^3^/ha^-1^) for each treatment in each block. Volume and spatial distribution of harvest residues in treatment plots in NC was shown to accurately match that of our original experimental design, which emulated target percentages of harvest residue retention proposed in BHGs [42]. Fritts et al. [42] calculated the following volumes (m^3^/ha^-1^) of harvest residues in each treatment in NC: NOBHGs = 20.65 ± 1.45; 15DISP = 40.80 ± 13.11; 15CLUS = 37.76 ± 9.42; 30DISP = 55.75 ± 12.49; 30CLUS = 55.17 ± 12.49; NOBIOHARV = 108.20 ± 20.05. Efficacy of treatment implementation in GA was less clear because windrowing altered spatial arrangement of harvest residues from that in the original experimental design.

### Quantifying vegetation

We quantified vegetation composition and structure in regenerating stands in NC in July, 2012–2014 and in GA in July, 2013 and 2014. We characterized vertical vegetation structure, maximum vegetation height, groundcover (i.e., cover less than 1 dm off the forest floor), and horizontal vegetation cover (i.e., cover 1 dm or greater off the forest floor) at systematically distributed vegetation plots in each treatment. In NC, we sampled nine plots/treatment in 2012 and 2013 and six plots/treatment in 2014. In GA, we sampled nine plots/treatment in 2013 and eight plots/treatment in 2014. At each vegetation plot, we established three, 10-m transects along which vegetation was measured at 10, 1-m increments (i.e., 30 total sampling points/vegetation plot). We oriented the first transect based on a random bearing and oriented the remaining two transects 120° to either side of the first transect [32]. As an index of vertical structure, we counted the number of times any vegetation (forb, grass, woody shrub/vine) touched any decimeter increment along a 2-m tall, 4.8-cm diameter rod at 30 sampling points [48]. We considered maximum vegetation height for each vegetation plot to be the average maximum decimeter increment (up to 2 m) at which we recorded a vegetative hit over the 30 sampling points. We recorded all groundcover types (bare ground, grass, and litter) that touched anywhere from the bottom through the first 1-dm increment of the rod. We recorded horizontal vegetation cover types (forb, grass, and woody shrub/vine) that touched anywhere above the 1-dm increment of the rod. We calculated percent cover of each groundcover and horizontal vegetation cover type at each vegetation plot by dividing the number of sampling points where the rod touched each groundcover or horizontal vegetation cover type by 30. For all vegetation metrics, we calculated means from pooled data across all plots in each treatment unit for each state and year.

### Statistical analysis

We used the most commonly detected species of breeding, early successional birds as focal species. Focal species included: 1) blue grosbeak (*Passerina caerulea*); 2) common yellowthroat (*Geothlypis trichas*); 3) eastern towhee (*Pipilo erythrophthalmus*); 4) field sparrow (*Spizella pusilla*); 5) indigo bunting (*Passerina cyanea*); 6) prairie warbler (*Setophaga discolor*); and 7) yellow-breasted chat (*Icteria virens*). We also considered Carolina wren (*Thryothorus ludovicianus*) a focal species because of documented relationships between this species and downed wood [26]. Of these focal species, we only included those with greater than 10 territories in treatments per breeding season in a single state in analyses. Among focal species, we also included northern bobwhite (*Colinus virginianus*), a regionally important early-successional gamebird with declining population trends [49]. Because northern bobwhite maintains loose territories that are difficult to determine [50], we did not delineate territories for the species and instead set our cutoff for analysis at ≥30 northern bobwhite detections in treatments per breeding season in a single state. To avoid biases induced by edge effects, we only included in our analyses detections and resultant territories recorded ≥25 m from all drainage ditches and adjacent forest edges. We also excluded detections recorded within logging decks in both states and within riparian forest stringers in the GA treatments.

We used territory density of each focal species and all focal species combined and species richness and the Shannon-Weaver index of diversity ([51]; hereafter “species diversity”) of all bird species combined as response variables to determine effects of harvest residue removal on the breeding bird community. We derived values for species diversity using the *diversity* function in the R package “vegan” [52]. We accounted for potential biases induced by variable treatment areas by defining territory densities as the number of territories per 40 ha (see Avian Sampling). We included relative abundance (i.e., count per treatment per year) as a response variable for northern bobwhite. To account for variation in survey effort, we standardized species richness, species diversity, and northern bobwhite relative abundance by dividing each by number of visits to each block per breeding season.

We tested for effects of harvest residue removal on each avian response variable using generalized linear models (GLMs). For the NC data, we first included a year x treatment interaction term and block as explanatory variables in each model. If we detected a significant year x treatment interaction, we consequently ran a model for each year separately and included treatment and block as independent, explanatory variables. Otherwise, we pooled data across years and included treatment, year, and block as independent, explanatory variables. We followed the same procedure for the GA avian analyses as we did for NC, but included volume of harvest residues (m^3^/ha^-1^) per treatment rather than treatment as an explanatory variable because windrowing at the GA blocks altered the distribution of downed wood in treatments such that we were unable to quantify the efficacy of the treatment classes. For GLMs, we assumed overdispersion when the residual deviance divided by the residual degrees of freedom was >1.0. If we detected overdispersion, we corrected for it by applying a negative binomial regression model [53]. We performed *post-hoc*, Tukey’s pair-wise comparisons of means among treatments and years using general linear hypothesis testing (*glht* function; single-step method) in the R package “multcomp” [54]. For GA avian analyses, we assessed model fit for models with significant effects of harvest residue volume on response variables using goodness-of-fit chi-squared tests.

We also examined effects of harvest residue removal on spatial and behavioral relative use of downed wood in treatments by the breeding bird community and focal species. Specifically, we included the count of all bird detections near, in, or on branches of piles and the count of all birds recorded foraging on the ground (i.e., not in vertical vegetation structure) and perching (excluding perching on vegetation) as response variables in the same models outlined above for the territory density analysis. Prior to analysis, we standardized counts of detections near, in, or on branches of piles and counts of birds recorded foraging on the ground or perching by dividing each by the number of visits to each block per year. We also calculated proportion of detections recorded near, in, or on branches of piles or among vegetation for each focal species for all years combined as a descriptor of relative use of harvest residues by focal species in regenerating stands.

We tested for effects of downed wood removal on the following vegetation characteristics for each state and year: 1) percent of each groundcover type (i.e., bare ground, grass, and litter); 2) percent of each horizontal vegetation cover type (i.e., forb, grass, and woody shrub/vine); 3) vertical vegetation structure; and 4) maximum vegetation height. For NC, we ran randomized complete block design analyses of variance (ANOVAs) with each vegetation characteristic as a response variable and treatment and block as fixed effects. For GA, we ran GLMs with each aforementioned vegetation characteristic as a response variable and volume of harvest residues (m^3^/ha^-1^) and block as independent, explanatory variables. We arcsine square-root transformed percentile variables, but only untransformed means and standard errors are reported. We performed *post-hoc*, pair-wise comparisons among treatments using Tukey’s Honest Significant Difference tests. We conducted all aforementioned analyses using statistical software program R [55]. We set α = 0.05.

## Results

Across three breeding seasons, we delineated 536 territories and detected 2,489 birds and 40 species in treatments (i.e., ≥25 m from edge) in NC and 654 territories, 4,024 birds and 63 species in treatments in GA; early-successional birds comprised a majority (~70%) of the species detected in treatments in both states (*see* S1 and S2 Tables). The following focal species met the minimum territory number for inclusion in analyses: 1) blue grosbeak (NC = 2012/2013; GA = 2013/2014); 2) Carolina wren (NC = 2012/2013; GA = 2013/2014); 3) common yellowthroat (NC and GA = 2013/2014); 4) eastern towhee (NC and GA = 2013/2014); 5) field sparrow (NC = 2013/2014); 6) indigo bunting (NC = 2013/2014; GA = 2012–2014); 7) prairie warbler (NC and GA = 2014); 8) northern bobwhite (NC and GA = 2013/2014); and 9) yellow-breasted chat (NC = 2014; GA = 2013/2014). Focal species accounted for 83% and 59% of total bird detections in the NC and GA treatments, respectively. Generalist species were more abundant in GA than NC. For example, northern cardinal (*Cardinalis cardinalis*) and northern mockingbird (*Mimus polyglottos*) collectively comprised approximately 15% of total bird detections in GA treatments.

Harvest residue removal on NC sites either had no effect on the early-successional bird community or effects lasted for only one year, and early-successional birds were unaffected by distribution of harvest residues (Table 1). Species diversity did not differ among treatments. In 2014, species richness was lower in the NOBHGs treatment than in the 15DISP treatment. Counts of individual birds detected on branches of piles were greater in 2012 than 2013. Counts of individual birds actively foraging on the ground (i.e., not foraging in vegetation) and perching on branches of piles were greater in 2012 than 2013 and 2014. Territory density of all focal species combined did not differ among treatments. In 2012, Carolina wren territory density was greater in the NOBIOHARV treatment than in all other treatments except 30CLUS. In 2013, common yellowthroat territory density was greater in the NOBIOHARV and 30DISP treatments than in the 15CLUS and 15DISP treatments. Northern bobwhite relative abundance was greater in the NOBHGs treatment than in the 15CLUS treatment. Territory density was greater in 2013 than in 2014 for indigo bunting and field sparrow.

**Table 1:** Mean (SE) species diversity and richness of all birds, count of all birds detected near, in, or on branches of piles, and count of all birds foraging on the ground (i.e., not in vegetation) and perching (excluding perching on vegetation) and mean (SE) focal species territory density per 40 ha in six woody biomass removal treatments in regenerating pine plantations, North Carolina (*n* = 4), 15 April– 15 July, 2012–2014. Treatments included: (1) no Biomass Harvesting Guidelines (NOBHGs); (2) 15% woody biomass retention in piles (15CLUS); (3) 15% woody biomass retention distributed evenly throughout the treatment unit (15DISP); (4) 30% woody biomass retention in piles (30CLUS); (5) 30% woody biomass retention distributed evenly throughout the treatment unit (30DISP); and (6) no woody biomass harvest (NOBIOHARV). We only included detections and resultant territories recorded ≥25 m from all drainage ditches and adjacent forest edge. We tested for treatment-level effects on response variables using GLMs with treatment, year, and block as independent, explanatory variables. Different letters indicate significantly different, pair-wise comparisons of treatment means at α = 0.05 level.

		Woody biomass removal treatments					
		NOBHGs	15CLUS	15DISP	30CLUS	30DISP	NOBIOHARV
All birds							
Community metrics							
Species diversity		0.21 (0.02)	0.19 (0.02)	0.20 (0.02)	0.18 (0.02)	0.20 (0.01)	0.19 (0.02)
Species richness ϒ							
	2012	0.52 (0.09)	0.46 (0.13)	0.44 (0.10)	0.38 (0.06)	0.56 (0.07)	0.48 (0.10)
	2013	1.06 (0.11)	0.93 (0.08)	1.18 (0.15)	0.90 (0.09)	1.05 (0.08)	0.96 (0.17)
	2014	1.09^**b**^ (0.08)	1.25^**ab**^ (0.07)	1.44^**a**^ (0.08)	1.25^**ab**^ (0.15)	1.31^**ab**^ (0.06)	1.12^**ab**^ (0.09)
Harvest residue-associated locations							
Near pile		0.51 (0.11)	0.55 (0.11)	0.34 (0.11)	0.38 (0.10)	0.30 (0.06)	0.40 (0.06)
In pile		0.06 (0.03)	0.08 (0.03)	0.11 (0.06)	0.22 (0.07)	0.17 (0.05)	0.20 (0.07)
On branch of pile ζ		0.97 (0.26)	0.93 (0.19)	0.79 (0.11)	0.93 (0.23)	0.89 (0.19)	1.32 (0.11)
Harvest residue-associated behaviors							
Foraging ζ		0.09 (0.04)	0.04 (0.03)	0.06 (0.04)	0.08 (0.04)	0.06 (0.02)	0.13 (0.04)
Perching ζ		1.10 (0.19)	1.11 (0.20)	1.31 (0.19)	1.29 (0.18)	1.32 (0.18)	1.29 (0.15)
Focal species							
Territory density/40 ha							
All focal species ζ							
	2012/2013/2014	19.96 (4.17)	22.65 (5.68)	26.46 (5.77)	28.13 (5.74)	31.49 (6.53)	31.14 (5.63)
Blue grosbeak							
	2012/2013	2.38 (1.00)	2.93 (0.59)	4.27 (0.77)	3.75 (0.73)	3.50 (0.97)	2.82 (0.68)
Carolina wren ϒ							
	2012	0^**b**^	0.80^**b**^ (0.80)	0.95^**b**^ (0.95)	2.33^**ab**^ (1.48)	0.80^**b**^ (0.80)	6.93^**a**^ (2.67)
	2013	0.92 (0.92)	0.26 (0.26)	0.95 (0.95)	0.77 (0.77)	2.94 (2.25)	3.02 (1.82)
	2014	N/A	N/A	N/A	N/A	N/A	N/A
Common yellowthroat ϒ							
	2012	N/A	N/A	N/A	N/A	N/A	N/A
	2013	11.15^**ab**^ (2.33)	8.64^**b**^ (2.16)	9.51^**b**^ (2.73)	11.64^**ab**^ (3.14)	16.13^**a**^ (2.32)	15.65^**a**^ (3.54)
	2014	8.54 (1.29)	9.31 (2.60)	11.67 (2.58)	10.44 (2.90)	11.64 (0.97)	8.99 (1.80)
Eastern towhee ϒ							
	2012	N/A	N/A	N/A	N/A	N/A	N/A
	2013	2.60 (0.92)	1.83 (1.83)	1.80 (1.04)	1.54 (0.89)	3.44 (1.41)	2.50 (1.54)
	2014	1.30 (0.75)	1.57 (0.91)	4.11 (2.06)	5.89 (2.35)	1.80 (1.04)	7.51 (4.13)
Field sparrow ζ							
	2013/2014	5.54 (1.85)	5.74 (1.41)	5.00 (1.37)	4.73 (0.99)	4.88 (1.07)	6.43 (1.55)
Indigo bunting ζ							
	2013/2014	4.48 (1.09)	4.85 (2.31)	4.89 (1.42)	7.53 (1.01)	8.27 (2.08)	7.27 (1.52)
Northern bobwhite^1^							
	2013/2014	0.34^**a**^ (0.10)	0.04^**b**^ (0.03)	0.20^**ab**^ (0.08)	0.26^**ab**^ (0.08)	0.20^**ab**^ (0.07)	0.26^**ab**^ (0.08)
Prairie warbler							
	2014	4.75 (1.64)	7.98 (4.08)	6.75 (1.52)	9.48 (1.96)	7.81 (2.24)	6.36 (1.81)
Yellow-breasted chat							
	2014	3.97 (1.32)	7.56 (2.39)	11.48 (3.06)	7.08 (1.84)	11.21 (1.09)	8.79 (0.90)

^1^Relative abundance rather than territory density used as response variable

ϒ = years analyzed separately when there was a significant year x treatment interaction

ζ = significant year effect

Similar to NC, harvest residue removal in GA had minimal or short-lived effects on the early-successional bird community (Table 2). Species diversity and richness was unaffected by volume of harvest residues. Counts of individual birds detected in piles increased with increasing volume of harvest residues in treatments (Pearson χ^2^ = 0.73) and increased from 2012 to 2013. Counts of individual birds actively foraging on the ground were greater in 2012 and 2014 than in 2013. In 2012, territory density of all focal species combined increased with increasing volume of harvest residues in treatments (Pearson χ^2^ = 0.68). Additionally, relative abundance of northern bobwhite and territory density of yellow-breasted chat increased with increasing volume of harvest residues in treatments (Pearson χ^2^ = 0.98 and 0.97, respectively). Indigo bunting territory density was lower in 2012 than in 2013 and 2014. Territory density was lower in 2013 than in 2014 for common yellowthroat, Carolina wren, eastern towhee, and yellow-breasted chat. Conversely, territory density for blue grosbeak decreased from 2013 to 2014. Relative abundance of northern bobwhite was lower in 2013 than 2014.

**Table 2:** Mean (SE) species diversity and species richness of all birds and focal species territory density per 40 ha in six woody biomass removal treatments in regenerating pine plantations, Glynn County (*n* = 3) and Chatham County (*n* = 1), Georgia, 15 April– 15 July, 2012–2014. Treatments included: (1) no Biomass Harvesting Guidelines (NOBHG); (2) 15% woody biomass retention in piles (15CLUS); (3) 15% woody biomass retention distributed evenly throughout the treatment unit (15DISP); (4) 30% woody biomass retention in piles (30CLUS); (5) 30% woody biomass retention distributed evenly throughout the treatment unit (30DISP); and (6) no woody biomass harvest (NOBIOHARV). We only included detections and resultant territories recorded ≥25 m from adjacent forest edge. We tested for effects of harvest residue removal using GLMs with harvest residue volume, year, and block as independent, explanatory variables.

		Harvest residue volume (m^3^/ha^-1^)		
		β	t	P
All birds				
Community metrics				
Species diversity ϒ				
	2012	0.0002	1.35	0.19
	2013	0.00007	1.50	0.15
	2014	0.00002	0.81	0.43
Species richness ϒ				
	2012	0.001	1.08	0.29
	2013	0.001	2.00	0.06
	2014	0.0008	1.22	0.24
Harvest residue-associated locations				
Near pile		0.0005	1.02	0.31
In pile ζ		0.001	2.87	<0.01
On branch of pile ϒ				
	2012	0.003	1.55	0.14
	2013	0.0007	0.31	0.76
Harvest residue-associated behaviors				
Foraging ζ		0.0007	2.42	0.02
Perching ϒ				
	2012	0.005	1.81	0.09
	2013	0.009	2.30	0.03
	2014	0.001	1.52	0.14
Focal species				
Territory density/40 ha				
All focal species ϒ				
	2012	0.006	2.25	0.04
	2013	0.014	1.89	0.07
	2014	0.013	1.62	0.12
Blue grosbeak ζ				
	2013/2014	0.0002	0.11	0.91
Carolina wren ζ				
	2013/2014	0.001	0.62	0.53
Common yellowthroat ζ				
	2013/2014	0.001	0.48	0.63
Eastern towhee ζ				
	2013/2014	-0.001	-0.47	0.64
Indigo bunting ζ				
	2012/2013/2014	0.002	1.53	0.13
Northern bobwhite^1^ ζ				
	2013/2014	0.002	3.63	<0.01
Prairie warbler				
	2014	0.002	0.81	0.43
Yellow-breasted chat ζ				
	2013/2014	0.003	2.01	0.05

^1^Relative abundance rather than territory density used as metric

ϒ = years were analyzed separately when there was a significant year x volume interaction

ζ = significant year effect

**Fig 1:**
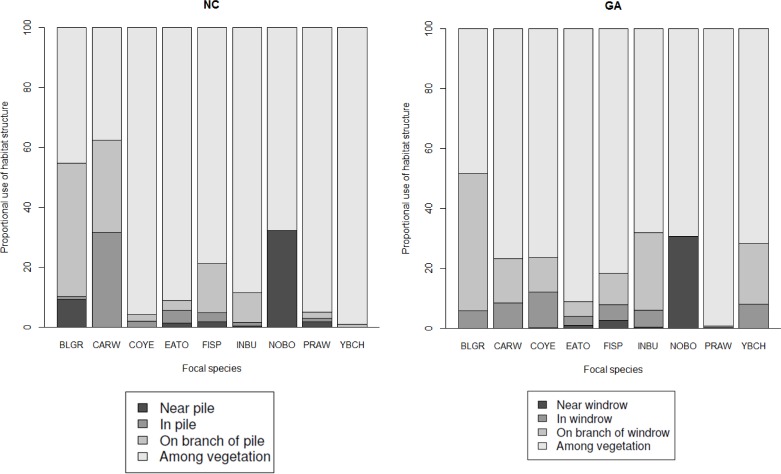
Relative use of habitat structure in NC (**a**) and GA (**b**), including harvest residue piles [i.e., downed wood; near, in, and on branches of piles (NC) or windrows (GA)] and vegetation, by focal species in regenerating pine plantations, North Carolina (*n* = 4) and Georgia (*n* = 4), 15 April– 15 July, 2012–2014. BLGR = blue grosbeak; CARW = Carolina wren; COYE = common yellowthroat; EATO = eastern towhee; FISP = field sparrow; INBU = indigo bunting; NOBO = northern bobwhite; PRAW = prairie warbler; and YBCH = yellow-breasted chat. See S. Appendices for scientific names of focal species.

In both states, most focal species used vegetation disproportionately to piles of downed wood (Fig 1**A** and 1**B**). In NC, >50% of blue grosbeak and Carolina wren detections were recorded near, in, or on branches of piles, but all other focal species were more frequently recorded using vegetation than downed wood piles. In GA, all focal species were more frequently recorded using vegetation than windrows. However, we documented more focal species using downed wood and recorded more individuals of focal species in piles of downed wood in GA than in NC. In NC, Carolina wren and field sparrow were detected near, on branches of, and especially in piles of downed wood more frequently than other focal species. In GA, northern bobwhite was detected near windrows far more than any other focal species. In addition to focal species, we documented many early successional, breeding bird species associating with piles of downed wood in regenerating stands (*see* S3 and S4 Tables).

In NC, we detected minimal effects of harvest residue removal on habitat characteristics, although some vegetation measures varied by treatments (Table 3). In 2012, woody shrub/vine cover was greater in the 30DSIP treatments than the 15DISP treatments. In 2013, vertical vegetation structure was greater in the 30CLUS treatments than the NOBIOHARV treatments. In 2014, grass cover was greater in the 30CLUS treatments than in the NOBIOHARV treatments, woody shrub/vine cover was greater in the NOBIOHARV and 30DISP treatments than in the 15CLUS treatment, and maximum vegetation height was greater in treatments with higher volumes of harvest residues than those with less.

**Table 3:** Mean (SE) of habitat characteristics in six woody biomass removal treatments in regenerating pine plantations, North Carolina (*n* = 4), July, 2012–2014. Treatments included: (1) no Biomass Harvesting Guidelines (NOBHGs); (2) 15% woody biomass retention in piles (15CLUS); (3) 15% woody biomass retention distributed evenly throughout the treatment unit (15DISP); (4) 30% woody biomass retention in piles (30CLUS); (5) 30% woody biomass retention distributed evenly throughout the treatment unit (30DISP); and (6) no woody biomass harvest (NOBIOHARV). We tested for treatment-level effects using randomized complete block design ANOVAs with each habitat characteristic as a response variable, treatment and block as explanatory variables. Different letters indicate significantly different, pair-wise comparisons of treatment means at α = 0.05 level.

		Woody biomass removal treatments					
Habitat characteristics		NOBHGs	15CLUS	15DISP	30CLUS	30DISP	NOBIOHARV
*Groundcover (%)*							
Bare ground							
	2012	53.24 (4.12)	53.24 (1.96)	53.43 (1.94)	50.28 (1.44)	49.26 (3.62)	47.50 (5.38)
	2013	37.22 (3.51)	31.76 (4.97)	33.36 (2.71)	29.63 (3.85)	31.96 (4.13)	30.19 (4.66)
	2014	35.39 (6.20)	28.50 (2.75)	21.86 (3.12)	29.17 (5.51)	30.72 (2.93)	24.72 (0.86)
Grass							
	2012	5.74 (2.35)	6.57 (1.46)	4.54 (2.45)	5.37 (1.79)	8.24 (3.07)	4.26 (1.37)
	2013	37.59 (2.96)	33.24 (2.05)	31.82 (4.68)	39.17 (3.21)	33.96 (5.54)	27.84 (3.56)
	2014	15.39 (1.93)	18.17 (1.22)	13.60 (2.13)	17.08 (1.72)	14.28 (1.67)	13.50 (1.76)
Litter							
	2012	21.94 (3.18)	21.11 (2.87)	24.26 (3.50)	22.13 (4.45)	20.19 (2.19)	27.04 (2.37)
	2013	13.61 (1.84)	19.54 (1.74)	19.14 (2.94)	14.91 (4.02)	15.01 (3.73)	25.43 (2.25)
	2014	39.61 (5.94)	42.78 (3.12)	54.61 (1.31)	47.64 (4.36)	45.33 (3.59)	46.86 (2.46)
*Vegetation cover (%)*							
Forb							
	2012	5.46 (1.77)	3.52 (1.59)	5.00 (1.80)	5.83 (1.96)	5.37 (1.36)	5.19 (1.74)
	2013	18.98 (3.81)	17.78 (1.87)	20.56 (4.16)	17.41 (3.54)	19.93 (2.03)	23.78 (0.99)
	2014	18.28 (2.77)	16.69 (1.47)	19.01 (3.01)	13.47 (2.76)	18.86 (3.52)	17.83 (3.82)
Grass							
	2012	7.96 (3.53)	7.69 (1.67)	6.02 (3.06)	7.78 (2.41)	11.20 (3.68)	6.11 (1.69)
	2013	56.11 (5.22)	54.26 (4.26)	54.41 (12.73)	60.65 (10.42)	54.84 (8.63)	46.82 (5.97)
	2014	63.86^**ab**^ (4.35)	65.03^**ab**^ (5.30)	57.04^**ab**^ (8.89)	66.67^**a**^ (2.05)	61.81^**ab**^ (8.40)	47.11^**b**^ (4.59)
Woody shrub/vine							
	2012	5.37^**ab**^ (1.07)	3.89^**ab**^ (1.11)	2.87^**b**^ (1.18)	4.35^**ab**^ (1.32)	6.57^**a**^ (1.65)	6.20^**ab**^ (1.07)
	2013	25.37 (1.96)	22.59 (5.09)	31.83 (9.52)	32.22 (8.23)	35.32 (7.33)	35.10 (7.33)
	2014	45.56^**ab**^ (3.18)	36.75^**b**^ (6.04)	48.43^**ab**^ (9.86)	54.31^**ab**^ (8.22)	57.25^**ab**^ (8.08)	63.42^**a**^ (5.15)
Vertical vegetation structure							
	2012	0.49 (0.14)	0.51 (0.16)	0.34 (0.10)	0.55 (0.17)	0.48 (0.09)	0.52 (0.18)
	2013	4.08^**ab**^ (0.29)	4.03^**ab**^ (0.35)	3.80^**ab**^ (0.41)	4.59^**a**^ (0.58)	4.46^**ab**^ (0.44)	3.67^**b**^ (0.38)
	2014	3.67 (0.18)	3.79 (0.09)	3.58 (0.36)	4.01 (0.28)	4.16 (0.24)	4.14 (0.13)
Maximum vegetation height							
	2012	0.59 (0.13)	0.57 (0.17)	0.40 (0.12)	0.65 (0.18)	0.61 (0.08)	0.61 (0.22)
	2013	5.57 (0.38)	5.70 (0.50)	5.24 (0.58)	6.23 (0.74)	6.20 (0.58)	5.46 (0.56)
	2014	7.39^**bc**^ (0.35)	6.91^**c**^ (0.14)	7.00^**bc**^ (0.45)	7.86^**abc**^ (0.39)	7.96^**ab**^ (0.51)	8.57^**a**^ (0.49)

In GA, most habitat characteristics were unaffected by harvest residue removal (Table 4). Grass groundcover (2013) decreased with increasing volume of harvest residues, whereas litter groundcover (2014) increased with increasing volume of harvest residues. In both 2013 and 2014, grass cover decreased with increasing volume of harvest residues. In 2014, woody shrub/vine cover increased with increasing volume of harvest residues. For both states, vegetation composition and structure increased through time, following typical successional trajectories for regenerating stands (Table 3).

**Table 4:** Effects of harvest residue removal on habitat characteristics in six woody biomass removal treatments within regenerating pine plantations, Glynn County (*n* = 3) and Chatham County (*n* = 1), Georgia, July, 2013 and 2014. We tested for effects of harvest residue removal using GLMs with each habitat characteristic as a response variable and harvest residue volume (m^3^/ha^-1^) and block as independent, explanatory variables. We set α = 0.05.

		Harvest residue volume (m^3^/ha^-1^)		
Habitat characteristics		β	t	P
*Groundcover (%)*				
Bare ground				
	2013	-0.001	-0.58	0.57
	2014	-0.001	-0.44	0.66
Grass				
	2013	-0.001	-2.30	0.03
	2014	-0.001	-1.79	0.09
Litter				
	2013	-0.001	-0.67	0.51
	2014	0.001	2.58	0.02
*Vegetative cover (%)*				
Forb				
	2013	0.001	0.12	0.91
	2014	0.001	0.77	0.45
Grass				
	2013	-0.001	-2.29	0.03
	2014	-0.001	-2.38	0.03
Woody shrub/vine				
	2013	0.001	1.53	0.14
	2014	0.001	2.60	0.02
Vertical vegetation structure				
	2013	0.001	0.79	0.44
	2014	-0.001	-0.87	0.40
Maximum vegetation height				
	2013	0.001	0.40	0.70
	2014	-0.001	-0.33	0.74

## Discussion

Our results suggest that the successional trajectory of vegetation structure and composition rather than volume or distribution of harvest residues following timber harvest is the primary driver of breeding, early-successional bird use of regenerating stands. Indeed, the early-successional bird community showed minimal response to woody biomass removal treatments or volume of retained harvest residues. However, many breeding bird species were associated with downed wood and vegetation in regenerating stands (*see also* S3 and S4 Tables). Further, harvest residues may provide important cover or foraging sites for some species of breeding birds in regenerating stands when vegetation largely is absent due to young stand age and effects of site preparation.

The paucity of literature on early-successional bird use of downed wood restricts comparisons to other studies [23]. However, Lohr et al. [27] showed that weak excavating and secondary-cavity-nesting species, Neotropical migrants, and eastern towhee all had fewer breeding territories on plots where downed wood was experimentally removed in mature (40–50 year old) loblolly pine forests of the southeastern United States. Similarly, we found species-specific, positive responses to harvest residue retention by some Neotropical migrants (i.e., common yellowthroat and yellow-breasted chat) in young loblolly pine forests of the same region. However, we documented no relationship between harvest residue removal and territory densities of eastern towhee. Overall, Lohr et al. [27] documented a greater avian response to downed wood removal in mature pine stands than we did in young, regenerating stands, which may be attributable to their analysis of guilds rather than species-specific metrics or inherent differences in relationships between birds, downed wood, and understory vegetation at different successional stages. For example, mature, commercial pine stands have greater canopy cover than regenerating clearcut stands and thus have less understory vegetation [56, 57], potentially making downed wood a more prominent habitat component on the forest floor in older pine forests.

Despite the fact that the early-successional bird community largely was unaffected by downed wood removal, our results indicate downed wood may be an important structural characteristic for some early-successional species. Although northern bobwhite was present in GA only when in-stand vegetation was well established (i.e., 2013 and 2014), relative abundance of the species increased with increasing volume of downed wood. In terms of documented associations with downed wood, northern bobwhite previously has only been listed as a “log rooster” [24]. Our results indicated northern bobwhite likely uses downed wood for cover in regenerating stands, suggesting that downed wood retention following forest harvests may benefit northern bobwhite using young clearcuts. In NC, Carolina wren territory density was greater in treatments with more downed wood retention than those with less, albeit when vegetation structure was minimal (2012); the species commonly associated with downed wood piles, verifying observations by Lanham and Guynn [24] and Hamel [26] that this wren is heavily associated with downed wood. Downed wood also may be used by some Neotropical migrant, early successional species, including common yellowthroat and yellow-breasted chat, for cover, food, and territory defense.

Based on comparisons of species responses between concurrent studies at some of the same research sites, breeding birds responded to harvest residue removal more than did winter birds (*see* [58]). Lohr et al. [27] recorded similar disparities between breeding and winter bird responses to down wood removal in mature, loblolly pine forests of the southeastern United States. Differences between breeding and winter bird response to harvest residue removal may be related to the suite of species occurring in each season or differences in the amount of downed wood necessary to meet foraging versus nesting requirements [23, 59]. Additionally, winter birds typically are non-territorial, and thus may be less strongly tied to particular habitat elements [27].

Our results support the notion inferred by previous studies that downed wood is used by birds for foraging and escape cover [27, 60]. Furthermore, we recorded several early successional bird species using downed wood that were previously not listed as doing so in the southeastern United States (*see* [24, 26]; S3 and S4 Tables). In GA, we detected more birds in piles (i.e., windrows) in areas with more overall harvest residue retention. Birds detected in windrows may have been using downed wood as cover, potentially in response to observer presence, and/or for gleaning invertebrate prey contained within windrows [27, 60]. For example, we often recorded Carolina wren in piles; the species uses downed wood for nesting and escape cover [26] and for the highly abundant arthropod prey base it harbors [61]. In GA, ground-foraging insectivores (e.g., eastern towhee) likely took advantage of abundant, downed-wood associated invertebrate prey near piles [62, 63]. Ground-foraging granivores (e.g., mourning dove, northern bobwhite) were more frequently detected near piles of downed wood than insectivorous focal species, potentially suggesting that the significant ground-foraging response to increasing volume of harvest residues in GA is related to locally abundant seed resources dispersed by flooding or wind-lodging against windrows [64]. Both insectivorous and granivorous, ground-foraging birds also may use downed wood for protective cover from aerial predators while feeding (e.g., diurnal raptors; [65]).

Downed wood may be especially important as habitat structure for early-successional birds in regenerating stands prior to significant revegetation (i.e., 0–1 year post-harvest). In 2012, territory density of all focal species combined was positively correlated with increasing harvest residue volumes in GA, which exhibited lower vegetation structure and composition relative to other years due to young stand age coupled with higher intensity site preparation (e.g., windrowing, broadcast herbicide treatments). In 2012, the NC blocks had the lowest vegetation structure compared to 2010–2011; meanwhile, detections of birds on branches of piles, ground-foraging, and perching were all greater in 2012 relative to later years. Downed wood provides perching platforms used by birds [60, 66]. Our findings suggest that birds in regenerating stands may select perching platforms based on height rather than substrate, using branches of downed wood until growing vegetation exceeds that height. Further, availability of perches on branches of downed wood in the absence of other structure may facilitate predator vigilance among breeding birds [27]. Similarly, availability of downed wood during early stand development may influence foraging opportunities for early-successional birds. For instance, insectivorous birds with flexible foraging strategies may center their feeding on ground-dwelling invertebrates harbored by downed wood in the absence of other structure during pre-vegetation establishment, and then shift to foliage-gleaning of phytophagous insects once vegetation becomes established in regenerating stands. However, most of the focal species established territories only after vegetation structure and composition (e.g., woody vegetation for shrub/scrub nesters) were well-established.

Differences in site preparation between study sites allowed us the unique opportunity to at least observationally explore potential variability in harvest residue retention and consequent avian response to woody biomass harvests following common silvicultural practices, namely shearing (NC) and windrowing (GA). Importantly, woody biomass harvests are predicted to predominantly occur in intensively managed pine stands [23], where implementation of chemical and/or mechanical site preparation practices to facilitate crop-tree survival and growth is nearly ubiquitous. In GA, windrowing had profound effects on the spatial arrangement of downed wood originally assigned to treatments in our experimental design and concentrated harvest residues into large piles. Meanwhile, shearing in NC had less influence on spatial arrangements of downed wood; as such, harvest residues were arranged similar to pre-mechanical site preparation conditions [42]. Thus, piles of downed wood were much smaller in NC compared to windrows in GA, which likely is why we detected far more birds “in piles” in GA. This finding may indicate that larger piles of downed wood are more often used by birds, likely for food and cover, compared to smaller ones in regenerating stands.

## Conclusion

Early-successional, breeding bird community metrics and territory densities mostly were unaffected by current levels of woody biomass harvest in intensively managed forests of the southeastern United States, suggesting that existing BHGs are potentially unnecessary with respect to breeding birds in the region. Further, redistribution of harvest residues by loggers for use by early-successional birds in regenerating stands may be futile in managed forests with intensive site preparation. Early-successional birds appeared to respond to successional changes in vegetation structure and composition more than to availability of harvest residues. However, several early successional bird species used harvest residues in some capacity, many of which were previously undocumented as doing so. As such, downed wood may be a relatively valuable habitat component following timber harvest in intensively managed forests. Current levels of woody biomass harvest leave considerable volumes of downed wood on the landscape [42, 67], yet technological advances in harvest machinery or increases in the market value of woody biomass could result in intensified removal of downed wood. If future woody biomass harvests intensify leading to a substantial decrease in harvest residue retention relative to the levels recorded in the current study, we recommend that breeding, early-successional bird response to woody biomass harvests be re-evaluated to inform and update BHGs. Additionally, studies spanning further along the successional timeline could shed new light on the interplay between harvest residues and vegetation and consequential avian response. However, density of most early-successional birds declines significantly after canopy-closure, when harvest residues availability is concurrently reduced due to decay [19, 21, 56, 68].

## Supporting Information

S1 Table Number and location of breeding birds observed in regenerating stands (*n* = 4) and surrounding edge, 15 April– 15 July, 2012–2014, Beaufort County, North Carolina. Regenerating stands and treatments therein were bordered by drainage ditches (~1 m wide) containing vegetation which was unaffected by site preparation and thus more developed than vegetation in treatments. Locations in treatments included: Interior = ≥ 50 m from drainage ditches and adjacent forest edge (AFE); Moderate = 25–50 m from drainage ditches and AFE; and Short = 1–25 m from drainage ditches and AFE. A logging road (~3.7 m wide) separated each side of most regenerating stands and adjacent forest stands, which typically fell into two age classes: 1) young (~10 years old); and 2) mature (~30 years old).
(PDF)

S2 Table Number and location of breeding birds observed in regenerating stands (*n* = 4) and surrounding edge, 15 April– 15 July, 2012–2014, Glynn and Chatham counties, Georgia. Regenerating stands and treatments therein were bordered by drainage ditches (~1 m wide) containing vegetation which was unaffected by site preparation and thus more developed than vegetation in treatments. Locations in treatments included: Interior = ≥ 50 m from drainage ditches and adjacent forest edge (AFE); Moderate = 25–50 m from drainage ditches and AFE; and Short = 1–25 m from drainage ditches and AFE. A logging road (~3.7 m wide) separated each side of most regenerating stands and adjacent forest stands, which typically fell into two age classes: 1) young (~10 years old); and 2) mature (~30 years old).
(PDF)

S3 Table Total counts of breeding birds detected near (within 1 m of pile), in, or on branches of piles of downed wood in regenerating stands (*n* = 4), 15 April– 15 July, 2012–2014, Beaufort County, North Carolina. (PDF)

S4 Table Total counts of breeding birds detected near (within 1 m of windrow), in, or on branches of windrows in regenerating stands (*n* = 4), 15 April– 15 July, 2012–2014, Glynn and Chatham counties, Georgia. (PDF)
