# Supplementary material for: Breeding, Early-Successional Bird Response to Forest Harvests for Bioenergy
Source: PLoS One. 2016 Oct 25;11(10):e0165070. doi: 10.1371/journal.pone.0165070 (PMC5079583; doi:10.1371/journal.pone.0165070)
Supplement: S1 Table — Regenerating stands and treatments therein were bordered by drainage ditches (~1 m wide) containing vegetation which was unaffected by site preparation and thus more developed than vegetation in treatments. Locations in treatments included: Interior = ≥ 50 m from drainage ditches and adjacent forest edge (AFE); Moderate = 25–50 m from drainage ditches and AFE; and Short = 1–25 m from drainage ditches and AFE. A logging road (~3.7 m wide) separated each side of most regenerating stands and adjacent forest stands, which typically fell into two age classes: 1) young (~10 years old); and 2) mature (~30 years old). (PDF) [file pone.0165070.s001.pdf]

S1 Table. Number and location of breeding birds observed in regenerating stands (n = 4) and surrounding edge, 15 April – 15 July, 2012–2014, Beaufort County, North Carolina. Regenerating stands and treatments therein were bordered by drainage ditches (~1 m wide) containing vegetation which was unaffected by site preparation and thus more developed than vegetation in treatments. Locations in treatments included: Interior = ≥50 m from drainage ditches and adjacent forest edge (AFE); Moderate = 25-50 m from drainage ditches and AFE; and Short = 1-25 m from drainage ditches and AFE. A logging road (~3.7 m wide) separated each side of most regenerating stands and adjacent forest stands, which typically fell into two age classes: 1) young (~10 years old); and 2) mature (~30 years old).

Common name	Scientific name	Interior	Moderate	Short	Drainage ditch	AFE (mature)	AFE (young)	Total
Acadian flycatcher	*Empidonax virescens*	0	0	0	0	4	4	8
American crow	*Corvus brachyrhynchos*	4	10	1	8	0	2	25
American goldfinch	*Spinus tristis*	2	1	1	9	1	3	17
American kestrel	*Falco sparverius*	2	0	2	0	0	1	5
Bald eagle	*Haliaeetus leucocephalus*	0	0	0	0	1	0	1
Barn swallow	*Hirundo rustica*	0	1	1	0	0	0	2
Black-and-white warbler	*Mniotilta varia*	0	0	0	0	6	1	7
Blue-gray gnatcatcher	*Polioptila caerulea*	0	1	0	5	35	1	41
Blue grosbeak	*Passerina caerulea*	73	95	128	172	7	30	505
Bluejay	*Cyanocitta cristata*	0	0	0	0	7	0	7
Brown thrasher	*Toxostoma rufum*	6	10	14	47	16	12	105
Brown-headed cowbird	*Molothrus ater*	11	19	28	22	7	23	110
Brown-headed nuthatch	*Sitta pusilla*	0	0	0	0	1	0	1
Carolina chickadee	*Poecile carolinensis*	2	2	0	9	13	2	28
Carolina wren	*Thryothorus ludovicianus*	33	32	64	35	124	74	358
Cedar waxwing	*Bombycilla cedrorum*	0	0	0	0	1	0	1
Chipping sparrow	*Spizella passerina*	0	0	1	0	2	2	5
Chuck-wills-widow	*Antrostomus carolinensis*	1	0	0	0	0	0	1
Common grackle	*Quiscalus quiscula*	0	0	0	8	1	2	11
Common nighthawk	*Chordeiles minor*	13	9	11	2	0	0	35
Common yellowthroat	*Geothlypis trichas*	318	181	187	206	42	94	1028
Cooper's hawk	*Accipiter cooperii*	0	0	1	0	0	0	1
Downy woodpecker	*Picoides pubescens*	0	0	0	0	9	0	9
Eastern bluebird	*Sialia sialis*	13	16	23	17	0	8	77

Eastern kingbird	*Tyrannus tyrannus*	29	20	61	86	10	15	221
Eastern meadowlark	*Sturnella magna*	0	0	0	1	0	0	1
Eastern phoebe	*Sayornis phoebe*	0	0	1	0	1	0	2
Eastern towhee	*Pipilo erythrophthalmus*	117	58	41	228	82	163	689
Eastern wood-pewee	*Contopus virens*	0	0	1	1	29	2	33
Field sparrow	*Spizella pusilla*	189	117	126	111	2	25	570
Gray catbird	*Dumetella carolinensis*	0	2	9	94	102	104	311
Great-crested flycatcher	*Myiarchus crinitus*	1	4	4	22	80	15	126
Hairy woodpecker	*Leuconotopicus villosus*	0	0	0	1	1	0	2
Hooded warbler	*Setophaga citrina*	0	0	0	0	1	0	1
Indigo bunting	*Passerina cyanea*	183	104	116	282	56	66	807
Killdeer	*Charadrius vociferus*	3	1	1	0	0	0	5
Mallard	*Anas platyrhynchos*	0	0	12	0	0	0	12
Mourning dove	*Zenaida macroura*	60	67	116	91	16	18	368
Northern bobwhite	*Colinus virginianus*	56	33	37	14	7	37	184
Northern cardinal	*Cardinalis cardinalis*	4	4	8	17	25	18	76
Northern flicker	*Colaptes auratus*	1	0	5	13	19	2	40
Northern mockingbird	*Mimus polyglottos*	10	26	22	42	3	6	109
Orchard oriole	*Icterus spurius*	10	5	12	46	16	4	93
Ovenbird	*Seiurus aurocapilla*	0	0	0	0	5	2	7
Palm warbler	*Setophaga palmarum*	0	0	1	0	0	0	1
Pine warbler	*Setophaga pinus*	0	0	16	1	21	0	38
Pileated woodpecker	*Hylatomus pileatus*	0	0	0	0	10	0	10
Prairie warbler	*Setophaga discolor*	197	51	49	204	56	220	777
Purple martin	*Progne subis*	0	0	0	20	0	0	20
Red-bellied woodpecker	*Melanerpes carolinus*	0	1	0	1	13	1	16
Red-eyed vireo	*Vireo olivaceus*	0	0	0	0	10	0	10
Red-headed woodpecker	*Melanerpes erythrocephalus*	0	1	4	0	20	3	28
Red-tailed hawk	*Buteo jamaicensis*	0	0	0	1	4	0	5
Ruby-throated hummingbird	*Archilochus colubris*	0	1	0	0	1	0	2
Savannah sparrow	*Passerculus sandwichensis*	2	2	1	2	0	0	7
Song Sparrow	*Melospiza melodia*	0	1	2	1	0	0	4
Sparrow spp.	n/a	0	0	8	0	0	0	8

Summer tanager	*Piranga rubra*	4	4	13	13	31	1	66
Swamp sparrow	*Melospiza georgiana*	3	0	2	0	0	0	5
Tufted titmouse	*Baeolophus bicolor*	1	0	0	1	14	1	17
Turkey vulture	*Cathartes aura*	3	0	6	0	0	1	10
White-eyed vireo	*Vireo griseus*	4	3	9	50	71	70	207
Wild turkey	*Meleagris gallopavo*	7	9	5	2	4	0	27
Wood thrush	*Hylocichla mustelina*	0	0	0	0	6	1	7
White-throated sparrow	*Zonotrichia albicollis*	0	0	2	1	1	0	4
Yellow-breasted chat	*Icteria virens*	198	43	52	266	62	162	783
Yellow-rumped warbler	*Setophaga coronata*	0	0	0	2	5	0	7
Yellow-throated warbler	*Setophaga dominica*	0	0	0	4	6	0	10
Total		1558	931	1204	2160	1067	1194	8114
