# Supplementary material for: Breeding, Early-Successional Bird Response to Forest Harvests for Bioenergy
Source: PLoS One. 2016 Oct 25;11(10):e0165070. doi: 10.1371/journal.pone.0165070 (PMC5079583; doi:10.1371/journal.pone.0165070)
Supplement: S2 Table — Regenerating stands and treatments therein were bordered by drainage ditches (~1 m wide) containing vegetation which was unaffected by site preparation and thus more developed than vegetation in treatments. Locations in treatments included: Interior = ≥ 50 m from drainage ditches and adjacent forest edge (AFE); Moderate = 25–50 m from drainage ditches and AFE; and Short = 1–25 m from drainage ditches and AFE. A logging road (~3.7 m wide) separated each side of most regenerating stands and adjacent forest stands, which typically fell into two age classes: 1) young (~10 years old); and 2) mature (~30 years old). (PDF) [file pone.0165070.s002.pdf]

S2 Table. Number and location of breeding birds observed in regenerating stands (*n* = 4) and surrounding edge, 15 April – 15 July, 2012–2014, Glynn and Chatham counties, Georgia. Regenerating stands and treatments therein were bordered by drainage ditches (~1 m wide) containing vegetation which was unaffected by site preparation and thus more developed than vegetation in treatments. Locations in treatments included: Interior = ≥50 m from drainage ditches and adjacent forest edge (AFE); Moderate = 25-50 m from drainage ditches and AFE; and Short = 1-25 m from drainage ditches and AFE. A logging road (~3.7 m wide) separated each side of most regenerating stands and adjacent forest stands, which typically fell into two age classes: 1) young (~10 years old); and 2) mature (~30 years old).

Common name	Scientific name	Interior	Moderate	Short	Riparian forest edge	Mature forest edge	Total
Acadian flycatcher	*Empidonax virescens*	0	0	0	8	3	11
American crow	*Corvus brachyrhynchos*	2	4	9	4	7	26
American kestrel	*Falco sparverius*	0	3	0	0	0	3
American robin	*Turdus migratorius*	0	5	6	1	0	12
Barn owl	*Tyto alba*	0	0	1	0	0	1
Barn swallow	*Hirundo rustica*	8	4	1	0	0	13
Black-and-white warbler	*Mniotilta varia*	0	0	0	0	3	3
Blue-gray gnatcatcher	*Polioptila caerulea*	15	6	8	115	48	192
Blue grosbeak	*Passerina caerulea*	145	85	158	74	31	493
Bluejay	*Cyanocitta cristata*	3	2	6	32	22	65
Bobolink	*Dolichonyx oryzivorus*	2	3	58	0	0	63
Brown thrasher	*Toxostoma rufum*	27	26	80	75	20	228
Brown-headed cowbird	*Molothrus ater*	2	4	6	2	1	15
Carolina chickadee	*Poecile carolinensis*	0	8	21	15	14	58
Carolina wren	*Thryothorus ludovicianus*	57	37	120	140	110	464
Chipping sparrow	*Spizella passerina*	6	4	10	1	0	21
Chuck-wills-widow	*Antrostomus carolinensis*	1	0	0	0	2	3
Cliff Swallow	*Petrochelidon pyrrhonota*	0	0	3	0	0	3
Common grackle	*Quiscalus quiscula*	0	4	7	28	23	62
Common nighthawk	*Chordeiles minor*	0	1	1	2	0	4
Common yellowthroat	*Geothlypis trichas*	238	115	203	53	59	668
Dark-eyed junco	*Junco hyemalis*	0	1	0	0	0	1
Downy woodpecker	*Picoides pubescens*	0	0	3	24	16	43
Eastern bluebird	*Sialia sialis*	46	41	46	0	0	133

Eastern kingbird	*Tyrannus tyrannus*	70	62	100	32	8	272
Eastern towhee	*Pipilo erythrophthalmus*	268	51	102	177	71	669
Eastern wood-pewee	*Contopus virens*	1	0	6	18	20	45
Field sparrow	*Spizella pusilla*	12	13	13	1	0	39
Fish crow	*Corvus ossifragus*	0	0	0	1	1	2
Grasshopper sparrow	*Ammodramus savannarum*	0	0	1	0	0	1
Gray catbird	*Dumetella carolinensis*	24	12	21	16	4	77
Great-crested flycatcher	*Myiarchus crinitus*	18	11	39	151	53	272
Great egret	*Ardea alba*	4	0	1	1	0	6
Green heron	*Butorides virescens*	2	3	3	0	0	8
Hairy woodpecker	*Leuconotopicus villosus*	0	0	1	19	4	24
Hooded warbler	*Setophaga citrina*	3	1	5	6	8	23
Indigo bunting	*Passerina cyanea*	356	215	453	237	57	1318
Kentucky warbler	*Geothlypis formosa*	0	0	0	1	1	2
Killdeer	*Charadrius vociferus*	0	0	1	0	0	1
Little blue heron	*Egretta caerulea*	4	0	4	2	0	10
Loggerhead shrike	*Lanius ludovicianus*	0	1	0	0	0	1
Mourning dove	*Zenaida macroura*	115	84	191	77	28	495
Northern bobwhite	*Colinus virginianus*	93	62	122	24	2	303
Northern cardinal	*Cardinalis cardinalis*	212	101	314	319	130	1076
Northern flicker	*Colaptes auratus*	0	0	2	15	4	21
Northern mockingbird	*Mimus polyglottos*	157	143	285	142	10	737
Northern parula	*Setophaga americana*	8	15	45	110	67	245
Orchard oriole	*Icterus spurius*	16	6	17	40	2	81
Ovenbird	*Seiurus aurocapilla*	0	0	0	1	0	1
Painted bunting	*Passerina ciris*	1	3	2	4	4	14
Pine warbler	*Setophaga pinus*	3	8	10	45	61	127
Pileated woodpecker	*Hylatomus pileatus*	6	2	17	32	22	79
Prairie warbler	*Setophaga discolor*	179	75	128	9	5	396
Red-bellied woodpecker	*Melanerpes carolinus*	16	4	17	128	61	226
Red-eyed vireo	*Vireo olivaceus*	3	2	10	80	36	131
Red-headed woodpecker	*Melanerpes erythrocephalus*	11	7	35	20	4	77
Red-shouldered hawk	*Buteo lineatus*	1	0	1	1	1	4

Red-tailed hawk	*Buteo jamaicensis*	1	0	13	4	1	19
Red-winged blackbird	*Agelaius phoeniceus*	1	0	2	0	1	4
Ruby-throated hummingbird	*Archilochus colubris*	0	0	1	0	0	1
Savannah sparrow	*Passerculus sandwichensis*	7	7	13	0	0	27
Snowy egret	*Egretta thula*	0	1	0	0	0	1
Song Sparrow	*Melospiza melodia*	1	0	0	0	0	1
Spotted sandpiper	*Actitis macularius*	0	0	1	0	0	1
Summer tanager	*Piranga rubra*	8	13	42	38	21	122
Swamp sparrow	*Melospiza georgiana*	3	0	1	0	1	5
Tufted titmouse	*Baeolophus bicolor*	6	1	19	18	18	62
Turkey vulture	*Cathartes aura*	0	0	0	0	1	1
Veery	*Catharus fuscescens*	1	1	1	0	0	3
Eastern whip-por-will	*Caprimulgus vociferus*	0	1	1	0	0	2
White-eyed vireo	*Vireo griseus*	165	87	137	188	87	664
Wild turkey	*Meleagris gallopavo*	1	2	11	2	5	21
Wood duck	*Aix sponsa*	0	0	0	4	0	4
Wood stork	*Mycteria americana*	0	1	2	0	0	3
Wood thrush	*Hylocichla mustelina*	0	1	2	0	2	5
White-throated sparrow	*Zonotrichia albicollis*	0	0	1	0	0	1
Yellow warbler	*Setophaga petechia*	0	1	3	0	0	4
Yellow-billed cuckoo	*Coccyzus americanus*	1	1	0	26	11	39
Yellow-breasted chat	*Icteria virens*	267	74	143	113	23	620
Yellow-throated vireo	*Vireo flavifrons*	1	0	3	36	33	73
Yellow-throated warbler	*Setophaga dominica*	3	3	13	15	14	48
Total		2599	1425	3044	2727	1241	11036
