# Supplementary material for: Breeding, Early-Successional Bird Response to Forest Harvests for Bioenergy
Source: PLoS One. 2016 Oct 25;11(10):e0165070. doi: 10.1371/journal.pone.0165070 (PMC5079583; doi:10.1371/journal.pone.0165070)
Supplement: S3 Table — (PDF) [file pone.0165070.s003.pdf]

S3 Table. Total counts of breeding birds detected near (within 1 m of pile), in, or on branches of piles of downed wood in regenerating stands (*n* = 4), 15 April – 15 July, 2012–2014, Beaufort County, North Carolina.

Common name	Scientific name	Near pile	In pile	On branch of pile	Total
American crow	*Corvus brachyrhynchos*	3	0	4	7
American kestrel	*Falco sparverius*	0	0	2	2
Barn swallow	*Hirundo rustica*	1	0	1	2
Blue grosbeak	*Passerina caerulea*	26	4	129	159
Brown thrasher	*Toxostoma rufum*	2	4	13	19
Brown-headed cowbird	*Molothrus ater*	16	0	28	44
Carolina wren	*Thryothorus ludovicianus*	0	48	41	89
Chipping sparrow	*Spizella passerina*	0	0	1	1
Chuck-wills-widow	*Antrostomus carolinensis*	0	0	1	1
Common grackle	*Quiscalus quiscula*	0	0	1	1
Common nighthawk	*Chordeiles minor*	16	0	4	20
Common yellowthroat	*Geothlypis trichas*	0	13	17	30
Eastern bluebird	*Sialia sialis*	4	0	44	48
Eastern kingbird	*Tyrannus tyrannus*	2	0	83	85
Eastern phoebe	*Sayornis phoebe*	0	0	1	1
Eastern towhee	*Pipilo erythrophthalmus*	3	9	9	21
Eastern wood-pewee	*Contopus virens*	0	0	1	1
Field sparrow	*Spizella pusilla*	8	13	72	93
Gray catbird	*Dumetella carolinensis*	4	0	6	10
Great-crested flycatcher	*Myiarchus crinitus*	1	0	9	10
Indigo bunting	*Passerina cyanea*	1	5	42	48
Mourning dove	*Zenaida macroura*	131	0	44	175
Northern bobwhite	*Colinus virginianus*	40	0	0	40
Northern cardinal	*Cardinalis cardinalis*	0	0	3	3
Northern flicker	*Colaptes auratus*	2	0	0	2
Northern mockingbird	*Mimus polyglottos*	1	2	36	39
Orchard oriole	*Icterus spurius*	0	1	6	7
Palm warbler	*Setophaga palmarum*	0	0	1	1
Pine warbler	*Setophaga pinus*	1	0	6	7

Prairie warbler	*Setophaga discolor*	5	4	6	15
Red-bellied woodpecker	*Melanerpes carolinus*	1	0	0	1
Red-headed woodpecker	*Melanerpes erythrocephalus*	4	0	5	9
Savannah sparrow	*Passerculus sandwichensis*	3	1	2	6
Song Sparrow	*Melospiza melodia*	0	0	2	2
Sparrow spp.	n/a	7	0	1	8
Summer tanager	*Piranga rubra*	1	0	9	10
Swamp sparrow	*Melospiza georgiana*	1	1	1	3
Turkey vulture	*Cathartes aura*	9	0	6	15
Wild turkey	*Meleagris gallopavo*	3	0	0	3
Yellow-breasted chat	*Icteria virens*	0	0	4	4
Total		296	105	641	1042
