# Supplementary material for: Breeding, Early-Successional Bird Response to Forest Harvests for Bioenergy
Source: PLoS One. 2016 Oct 25;11(10):e0165070. doi: 10.1371/journal.pone.0165070 (PMC5079583; doi:10.1371/journal.pone.0165070)
Supplement: S4 Table — (PDF) [file pone.0165070.s004.pdf]

S4 Table. Total counts of breeding birds detected near (within 1 m of windrow), in, or on branches of windrows in regenerating stands (n = 4), 15 April – 15 July, 2012–2014, Glynn and Chatham counties, Georgia.

Common name	Scientific name	Near windrow	In windrow	On branch of windrow	Total
American robin	*Turdus migratorius*	0	0	1	1
Blue-gray gnatcatcher	*Polioptila caerulea*	0	2	0	2
Blue grosbeak	*Passerina caerulea*	4	23	183	210
Bluejay	*Cyanocitta cristata*	0	0	2	2
Bobolink	*Dolichonyx oryzivorus*	0	0	63	63
Brown thrasher	*Toxostoma rufum*	1	20	38	59
Brown-headed cowbird	*Molothrus ater*	3	0	1	4
Carolina chickadee	*Poecile carolinensis*	0	0	4	4
Carolina wren	*Thryothorus ludovicianus*	2	18	31	51
Common grackle	*Quiscalus quiscula*	2	0	3	5
Common nighthawk	*Chordeiles minor*	2	0	0	2
Common yellowthroat	*Geothlypis trichas*	1	66	67	134
Eastern bluebird	*Sialia sialis*	0	0	76	76
Eastern kingbird	*Tyrannus tyrannus*	0	1	49	50
Eastern towhee	*Pipilo erythrophthalmus*	7	13	21	41
Field sparrow	*Spizella pusilla*	1	2	4	7
Great-crested flycatcher	*Myiarchus crinitus*	0	0	9	9
Great egret	*Ardea alba*	3	0	1	4
Green heron	*Butorides virescens*	0	0	7	7
Hooded warbler	*Setophaga citrina*	0	0	1	1
Indigo bunting	*Passerina cyanea*	4	55	256	315
Little blue heron	*Egretta caerulea*	3	0	1	4
Mourning dove	*Zenaida macroura*	157	4	37	198
Northern bobwhite	*Colinus virginianus*	88	0	0	88
Northern cardinal	*Cardinalis cardinalis*	9	64	90	163
Northern mockingbird	*Mimus polyglottos*	14	36	273	323
Orchard oriole	*Icterus spurius*	1	7	17	25
Painted bunting	*Passerina ciris*	0	0	2	2
Prairie warbler	*Setophaga discolor*	0	1	2	3

Red-bellied woodpecker	*Melanerpes carolinus*	0	0	3	3
Red-eyed vireo	*Vireo olivaceus*	0	0	2	2
Red-headed woodpecker	*Melanerpes erythrocephalus*	0	1	2	3
Savannah sparrow	*Passerculus sandwichensis*	7	1	12	20
Snowy egret	*Egretta thula*	1	0	0	1
Spotted sandpiper	*Actitis macularius*	1	0	0	1
Summer tanager	*Piranga rubra*	1	4	28	33
Tufted titmouse	*Baeolophus bicolor*	0	2	1	3
White-eyed vireo	*Vireo griseus*	0	22	3	25
Wild turkey	*Meleagris gallopavo*	11	0	0	11
Wood stork	*Mycteria americana*	2	0	0	2
Yellow-breasted chat	*Icteria virens*	0	39	99	138
Yellow-billed cuckoo	*Coccyzus americanus*	0	1	0	1
Yellow-throated warbler	*Setophaga dominica*	0	1	7	8
Total		325	383	1396	2104
